# Cholesterol Dysmetabolism in Alzheimer’s Disease: A Starring Role for Astrocytes?

**DOI:** 10.3390/antiox10121890

**Published:** 2021-11-26

**Authors:** Erica Staurenghi, Serena Giannelli, Gabriella Testa, Barbara Sottero, Gabriella Leonarduzzi, Paola Gamba

**Affiliations:** Department of Clinical and Biological Sciences, University of Turin, Orbassano, 10043 Turin, Italy; erica.staurenghi@unito.it (E.S.); serena.giannelli@unito.it (S.G.); gabriella.testa@unito.it (G.T.); barbara.sottero@unito.it (B.S.); gabriella.leonarduzzi@unito.it (G.L.)

**Keywords:** Alzheimer’s disease, astrocytes, astrocyte reactivity, cholesterol, oxysterols, neuroinflammation, neurodegeneration, oxidative stress

## Abstract

In recent decades, the impairment of cholesterol metabolism in the pathogenesis of Alzheimer’s disease (AD) has been intensively investigated, and it has been recognized to affect amyloid β (Aβ) production and clearance, tau phosphorylation, neuroinflammation and degeneration. In particular, the key role of cholesterol oxidation products, named oxysterols, has emerged. Brain cholesterol metabolism is independent from that of peripheral tissues and it must be preserved in order to guarantee cerebral functions. Among the cells that help maintain brain cholesterol homeostasis, astrocytes play a starring role since they deliver de novo synthesized cholesterol to neurons. In addition, other physiological roles of astrocytes are to modulate synaptic transmission and plasticity and support neurons providing energy. In the AD brain, astrocytes undergo significant morphological and functional changes that contribute to AD onset and development. However, the extent of this contribution and the role played by oxysterols are still unclear. Here we review the current understanding of the physiological role exerted by astrocytes in the brain and their contribution to AD pathogenesis. In particular, we focus on the impact of cholesterol dysmetabolism on astrocyte functions suggesting new potential approaches to develop therapeutic strategies aimed at counteracting AD development.

## 1. Introduction

Alzheimer’s disease (AD) is the primary cause of dementia among the elderly, characterized by the gradual loss of memory and cognitive skills needed for day-to-day activities, and it is ranked as the fifth leading cause of death globally [1].

AD development is triggered by several events, including oxidative stress, inflammation, amyloid β (Aβ) plaque and neurofibrillary tangle (NFT) formation, causing neurodegeneration. All these events are interconnected in a self-sustaining circle exacerbating neuronal damage. The resulting cell death amplifies the inflammatory molecule release in the brain, favoring Aβ plaque and NFT formation, that triggers further inflammatory response and neurodegeneration [2]. Several cell types actively participate in this complex chain of events; however, the contribution of non-neuronal cells to the functional deficiencies that occur in AD remains largely unexplored.

In physiological conditions, astrocytes play a starring role in the brain since they contribute to the maintenance of glutamate, ion and water homeostasis, and they protect from oxidative/nitrosative stress. Moreover, they modulate synaptic transmission and plasticity and support neurons providing energy substrates and producing cholesterol. In response to non-physiological conditions, such as ischemia and infections, astrocytes undergo morphological, molecular, and functional changes, which together are referred to as astrocyte reactivity [3,4]. It is known that alterations in astrocytic functions contribute to AD pathogenesis, but the extent of this contribution is currently mostly unclear.

Among the risk factors that are understood to influence the occurrence of AD, dysregulation of cholesterol homeostasis in the brain has gained ground in the last decades [5] since hypercholesterolemia and the presence of the *ε4* allele of apolipoprotein E (ApoE) were discovered to enhance the risk of developing AD [6,7]. Further, a metabolomic and transcriptomic study [8], as well as a meta-analysis [9], have confirmed the association of AD with abnormalities in cholesterol biosynthesis and catabolism.

In normal conditions, highly controlled mechanisms exist to regulate and keep stable brain cholesterol steady-state levels, essential to maintain neuronal functions. In particular, since blood cholesterol cannot cross the blood brain barrier (BBB), excess cholesterol can be oxidized into more hydrophilic metabolites (i.e., oxysterols), in particular into 24-hydroxycholesterol (24-OHC), that exits the brain by diffusing through the BBB [10]. In AD brains, different oxysterols of enzymatic and non-enzymatic origin are generated and accumulate in toxic amounts, mainly because of the disruption of BBB integrity [11], playing a crucial role in AD development, particularly by enhancing oxidative stress and inflammation, causing neurodegeneration.

In this review, we focus on cholesterol metabolism in the brain and its alterations in AD. In particular, we summarize the current knowledge on the impact of cholesterol dysmetabolism on astrocytes, a field that so far remains largely unexplored but that could be relevant for the development of new therapeutic strategies aimed at counteracting AD onset and progression.

## 2. Astrocyte Functions and Their Contribution to Alzheimer’s Disease

### 2.1. The “Stars” of the Brain: The Key Physiological Roles of Astrocytes

Glial cells were originally described by Rudolf Virchow, who coined the term “glia” referring to brain cells that are not neurons and form a kind of “glue” [12]. Different types of glial cells were later identified, including astroglia, with the typical stellate shape, microglia, and oligodendroglia.

At first, astrocytes, based on their morphology and anatomical location, were classified into two main groups: protoplasmic and fibrous astrocytes. Protoplasmic astrocytes are localized in the cortical grey matter (cortical layers II-IV) and their morphology is characterized by highly branched processes that reach the pre- and post-synaptic terminals of neurons. Fibrous astrocytes, instead, are localized in the white matter along the neuronal axons; they are characterized by long and less branched processes and their main role is likely to provide metabolic support through contacts with blood vessels [13,14]. However, this classification is highly simplified. Astrocytes are actually much more heterogeneous from both the morphological and functional point of view, and their heterogeneity has not yet completely emerged [15]. For instance, additional specialized populations of astrocytes are present in the human brain, such as interlaminar astrocytes and varicose projection astrocytes [16]. Interlaminar astrocytes are primate-specific and located in cortical layer I; they are characterized by long processes that reach cortical layers III-IV, suggesting a potential function in long-distance intra-cortical communication [17]. Varicose projection astrocytes are present in cortical layers V-VI and are specific to humans and higher-order primates; they have long processes characterized by varicosities that terminate in the neuropil or contact blood vessels [17]. Moreover, human astrocytes are substantially more complex compared to rodent astrocytes; for instance, human protoplasmic astrocytes have more and longer processes, resulting in an increased cell volume and number of covered synapses. Furthermore, human fibrous astrocytes have been observed to be larger than rodent ones. Interestingly, this is thought to be one of the reasons for human brain complexity [17,18]. Transcriptomic analysis has highlighted marked differences between human and rodent astrocytes, such as differences in the expression levels of genes involved in metabolism and immune defense [18,19]. Furthermore, human astrocytes have also been shown to be more susceptible to oxidative stress, to have a lower mitochondrial respiration rate and different calcium response properties, and to react differently under hypoxia and inflammatory conditions [18,19]. Hence, these data have highlighted the importance of improved characterization of human astrocytes in order to deepen understanding of the basis of neurological disorders and to facilitate the clinical translatability of experimental therapeutic approaches.

Nowadays astrocytes are no longer considered only a structural element. In the last two decades, many studies have highlighted their involvement in essential central nervous system (CNS) functions, including neurogenesis, synaptic activity, blood flow regulation, ion and water homeostasis, energy metabolism, and immune response. Astrocytes have been shown to promote post-natal synaptogenesis [20] and to phagocytize unnecessary synapses in the adult hippocampus [21], both crucial functions for neural circuit maturation and plasticity. Concerning synaptic activity, astrocytes contact both the pre-synaptic axon terminal and post-synaptic dendritic spines, creating the so-called “tripartite synapse”. As a result of these connections, astrocytes can sense neuronal activity and in turn modulate synaptic transmission and plasticity, for instance by the re-uptake of glutamate from the synaptic cleft through glutamate transporters [22,23,24] or by releasing gliotransmitters (e.g., glutamate, ATP, and D-serine) [25,26,27].

Astrocytic perivascular processes are structural components of the neurovascular unit (NVU) that consists of different cell types including endothelial cells, vascular smooth muscle cells, pericytes, and astrocytes. One of the key functions of the NVU is “neurovascular coupling”, consisting in the regulation of the cerebral blood flow in response to local neuronal activity in order to provide enough oxygen and nutrients to neurons basing on their metabolic needs. Although data about the role of astrocytes in the regulation of the arteriolar tone are still controversial [28], studies have demonstrated that astrocytes mediate neurovascular coupling in the capillaries, regulating pericyte contraction/relaxation through the release of molecules such as arachidonic acid and prostaglandin E2 [29,30]. In addition, through the surface water channels aquaporins, astrocytes control the water flux between brain and bloodstream [31], as well as the clearance of toxic solutes, such as Aβ peptides [32].

Another important function of astrocytes is to support neurons providing energy substrates and producing cholesterol. Indeed, astrocytes provide energy by storing glucose in the form of glycogen and by delivering lactate through the lactate shuttle (glucose conversion to lactate, that is then taken up by neurons) in response to glutamate released from neurons [33,34]. The role of astrocytes in brain cholesterol homeostasis, as well as their involvement in some pathological changes related to AD, are reported in the next Sections.

### 2.2. Astrocyte Reactivity in Alzheimer’s Disease

#### 2.2.1. Astrocyte Reactivity and Its Complexity

Changes in glial cells associated with CNS diseases were observed as early as the 19th century [12] and evidence supporting astrocyte involvement in neurological disorders has increased over the years. Astrocytes undergo morphological, molecular, and functional changes in response to non-physiological conditions (e.g., trauma, ischemia, infections, and neurodegeneration): this wide range of changes has been defined with many terms, but the most used is “astrocyte reactivity” [3]. For a long time, reactive astrocytes have been identified by two main features, which are common to different pathologies: morphological changes and glial fibrillary acidic protein (GFAP) immunoreactivity. Changes in astrocyte morphology are due to cytoskeletal reorganization; they range from soma and process hypertrophy to ramification or polarization of processes toward a lesion or glial scar formation [35,36]. Concerning GFAP, it is a key constituent of intermediate filaments in astrocytes and the most widely used marker of astrocyte reactivity. GFAP immunoreactivity characterizes astrocytes in many CNS disorders, including AD [4,37,38,39]; however, it does not necessarily characterize all reactive astrocytes and its up-regulation varies depending on the pathology [3]. Although astrocyte reactivity has so far been considered mainly a process aimed at resolving tissue damage, the functional consequences on astrocytes are still controversial and studies have pointed out that the impact on surrounding cells might be beneficial or detrimental [3,40,41,42,43]. Indeed, astrocyte reactivity is a very heterogeneous response affected not only by the triggering stimulus, but also by the intrinsic features of the astrocytic population, the surrounding microenvironment, the CNS region, and by the distance to the injury core; this results in highly variable changes in terms of morphology, signaling pathways activation, molecular marker up-/down-regulation, as well as functional changes [3]. Transcriptomic analysis of reactive astrocytes showed that tens of genes are up- or down-regulated in lipopolysaccharide (LPS) and in middle cerebral artery occlusion (MCAO) mouse models [4], in AD animal models and in human AD brains [44,45], as well as in many other pathologies [3]. In particular, Zamanian and colleagues showed that at least 50% of the transcriptomic changes observed in reactive astrocytes depends on the specific injury [4]. Interestingly, a consensus statement has recently been published with the aim of clarifying nomenclature and definitions, as well as providing a critical evaluation of the most used reactive astrocyte markers. The authors recommended the evaluation of various potential markers of astrocyte reactivity in addition to GFAP and the assessment of key astrocytic functions (glutamate uptake, metabolic functions, and production of neurotrophic factors or cytokines) in order to determine whether astrocytes are actually reactive and which kind of phenotype they acquire [46].

#### 2.2.2. The Main Outcomes of Astrocyte Reactivity in Alzheimer’s Disease

Astrocyte reactivity is not an exclusive feature of AD, but it is considered to play a crucial role in this pathology since it is significantly associated with the presence of amyloid plaques [40]. The number of GFAP immunoreactive astrocytes has been shown to markedly increase in AD brains; in particular, they cluster around amyloid plaques, surrounding them with their processes [47,48,49,50]. Reactive astrocytes have also been shown to surround extracellular NFTs and their presence positively correlates with tangle burden [14,51]; moreover, tau accumulation in astrocytes of the hippocampus has been shown to induce neuronal dysfunction and memory deficits [52]. An interesting post mortem histopathological analysis revealed that the number of reactive astrocytes increase in the brain of demented AD subjects but not in the brain of the so-called “mismatches”, non-demented individuals whose post mortem examination showed significant amounts of amyloid plaques and NFTs [47]. This and other studies pointed out that astrocyte reactivity is likely one of the mediators of impaired cognition [47,53].

Due to the many key roles that astrocytes play in the CNS, astrocyte reactivity has several functional implications, many of which are still controversial. One of the main controversies concerns Aβ plaques, since it is not clear whether reactive astrocytes surrounding plaques contribute to their formation or clearance. In support of the first hypothesis, up-regulation of amyloid precursor protein (APP) and/or β-site APP cleaving enzyme 1 (BACE1) has been observed in reactive astrocytes (GFAP+) in 5XFAD and Tg2576 transgenic mice [54,55], as well as in human AD hippocampus and cortex [54,56]. Furthermore, astrocytes have also been shown to promote the amyloidogenic processing of APP, by up-regulating APP and/or BACE1 protein levels, in response to different stimuli such as inflammatory stimuli [57,58], chronic brain injury [54], or Aβ itself [55,59]. On the other hand, post mortem histopathological studies have highlighted the presence of Aβ within reactive astrocytes in human AD brains [60,61], suggesting the potential involvement of these cells in Aβ clearance. Astrocytes can, indeed, clear Aβ by phagocytosis [62,63,64] or by releasing Aβ-degrading enzymes, such as insulin degrading enzyme or matrix metalloproteinases [65,66]. In particular, ApoE and two ApoE receptors, namely the low-density lipoprotein receptor (LDLR) and the LDLR-related protein 1 (LRP1), have been shown to play a major role in Aβ degradation mediated by astrocytes (see Paragraph 3.3) [66,67,68,69]. However, another hypothesis explaining Aβ accumulation in AD astrocytes is that it could be the result of impaired lysosomal function [64,70]; in support of this, the stimulation of lysosomal biogenesis in astrocytes has been shown to reduce amyloid plaque load in the hippocampus of APP/PS1 transgenic mice and in vitro [71,72].

Astrocyte reactivity also potentiates secondary injury mechanisms, including inflammation, oxidative stress, and glutamate excitotoxicity, which facilitate neurodegeneration.

Oxidative stress and inflammation represent two main driving forces leading to the progression of AD, and astrocytes appear to be involved in both processes [73]. Astrocyte reactivity has been shown to impair the astrocyte secretion profile. For instance, the transcriptional analysis of astrocytes from APP/PS1 mice has shown an up-regulation of inflammatory genes and a reduced expression of genes involved in neuronal support [44]. Moreover, astrocytes from 5XFAD mice showed a reduced ability to support neuronal growth, an increase in interleukin 6 (IL-6) expression, and a neurotoxic effect [74]. Recently, an enrichment in genes involved in inflammation also emerged from the transcriptomic analysis of astrocytes from human AD brains [75]. Furthermore, several in vitro studies have shown that astrocytes can mediate neurotoxic or synaptotoxic effects by releasing pro-inflammatory cytokines, microvesicles, and other molecules in response to Aβ [64,76,77]. Oxysterols present in mild or severe AD brains also promote astrocyte reactivity by up-regulating some reactive astrocyte markers, including lipocalin-2 (Lcn2), and determining the release of several mediators that affect neuronal health and synapses [78]. In addition, inflammation itself can affect the astrocyte secretion profile [79]; for instance, IL-1α, tumor necrosis factor α (TNFα) and complement component 1, subcomponent q (C1q), released by activated microglia, have been shown to induce a reactive phenotype in astrocytes, that consequently lose their ability to promote synaptogenesis and neuronal survival inducing, instead, neuronal death [40].

It is known that astrocytes can promote and maintain the state of oxidative stress over time in pathological conditions, producing reactive oxygen species (ROS) and reactive nitrogen species (RNS), and, conversely, oxidative stress can lead to astrocyte defects [80,81]. Moreover, growing data suggests that oxidative stress can trigger astrocyte reactivity and that ROS/RNS production is increased in reactive astrocytes [81]. For instance, LPS has been shown to increase GFAP expression in primary astrocytes by inducing the production of nitric oxide (NO), one of the main RNS [82]. The injection into rat brains of the mitochondrial respiratory chain blocker rotenone induced astrocyte reactivity, stimulated NO production, and decreased the levels of glutathione (GSH), the major antioxidant in the CNS; data were confirmed in vitro treating C6 glioma cells with rotenone, showing increased GFAP synthesis and ROS/NO production, as well as impaired mitochondrial activity and DNA damage [83]. Treatment with hydrogen peroxide (H_2_O_2_) also induced GFAP up-regulation and increased pro-inflammatory cytokine release by astrocytes [84]. Furthermore, other stimuli able to induce astrocyte reactivity, such as Aβ or pro-inflammatory cytokines, have been shown to induce oxidative stress. Indeed, it has been demonstrated in rat astrocytes that Aβ favours the influx of intracellular free calcium from the extracellular space thus inducing mitochondrial dysfunction, characterized by mitochondrial depolarization, increased conductance, and opening of the mitochondrial transition pores [85]. Calcium signals induced by Aβ peptides have been shown to be also responsible for reduction of the antioxidant GSH in astrocytes [86]. Moreover, a prolonged incubation with aggregated Aβ, but not monomeric Aβ, inhibits GSH release via ATP-binding cassette transporter C1 (ABCC1) in astrocytes; in contrast, in the early stages of AD, less aggregated Aβ increases GSH release from astrocytes and provides temporary protection from oxidative stress [87]. Indeed, astrocytes may also play a central role in protecting neurons from oxidative damage since, in response to harmful molecular stimuli, they activate antioxidant enzymes and increase GSH levels [88]. Current evidence also suggests that oxidative stress and inflammation in astrocytes might be modulated by autophagy; in particular, dysfunctional autophagy in astrocytes is involved in oxidative stress and inflammation in age-related neurodegenerative diseases [89]. Autophagy inhibition decreased H_2_O_2_-induced astrocytic death, demonstrating the potential regulatory action of autophagy on astrocyte oxidative stress [90].

Growing evidence suggests that astrocyte reactivity affects the ability of astrocytes to modulate neuronal synaptic transmission, leading to excitotoxicity. One of the proposed mechanisms is the impairment of glutamate uptake. The reduction of glutamate transporter 1 (GLT-1) levels in reactive astrocytes surrounding plaques, and the increased glutamate concentration in the synaptic cleft, have been observed in APP/PS1 mice [91]. The partial loss of GLT-1 in the same AD mouse model (GLT-1^+/−^) led to the early appearance of spatial memory deficits [92]. Furthermore, the activation of human astrocytes with IL-1β was able to induce ROS production and to down-regulate glutamate uptake [93]. Interestingly, a post mortem histopathological study showed higher levels of astrocytic GLT-1 in the entorhinal cortex of non-demented subjects with AD brain pathology compared to AD subjects with dementia [94], suggesting that GLT-1 could contribute to preserving cognitive functions.

Transcriptomic studies are a powerful tool for analyzing the outcome of astrocyte reactivity on neurons, another aspect that still needs to be clarified. RNA sequencing of astrocytes from both mouse and human brains showed that transcriptional changes of aging astrocytes are region-specific [95] and very similar to those observed in neuroinflammatory reactive astrocytes [96,97]; this suggests that even normal aging could be a trigger of astrocyte reactivity. For instance, it has been shown that aged murine astrocytes up-regulate genes involved in synapse elimination and down-regulate genes responsible for the synthesis of cholesterol [96], a critical molecule for synapse formation and function [98].

**Figure 1:**
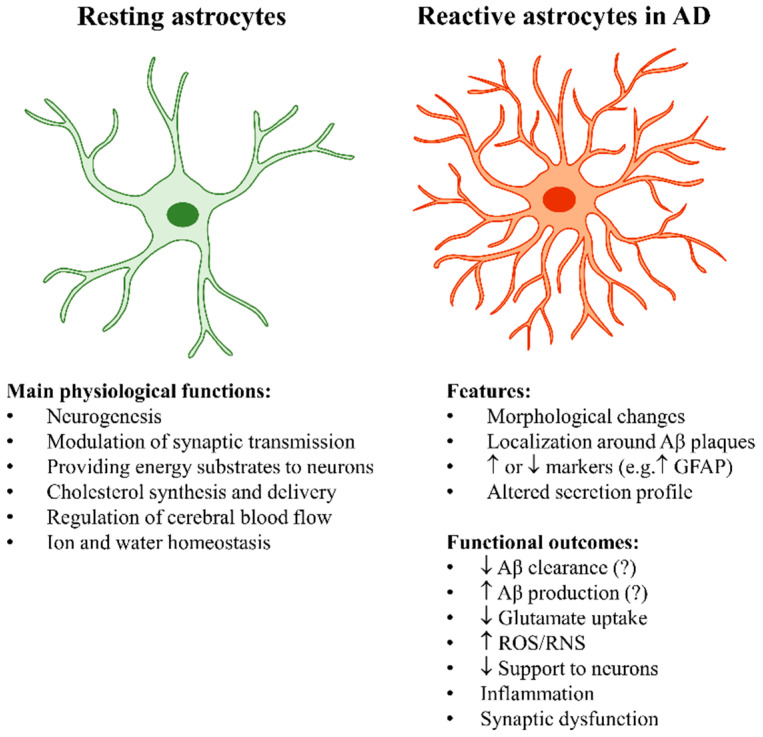
Main physiological functions of astrocytes and features of reactive astrocytes in AD. Abbreviations: Aβ, amyloid β; GFAP, glial fibrillary acidic protein; ROS, reactive oxygen species; RNS, reactive nitrogen species.

Those reported above are just some of the astrocytic functions affected by reactivity [3,14] (Figure 1). Conflicting data concerning the functional implications of astrocyte reactivity are likely due to the great variability of phenotypes that astrocytes can acquire. Overall, the transcriptomic studies mentioned above suggest that AD astrocytes are characterized by both loss of neuroprotective functions and gain of toxic functions.

## 3. The Involvement of Astrocytes in Brain Cholesterol Dysmetabolism

### 3.1. Astrocyte-Neuron Interplay in Brain Cholesterol Metabolism

In addition to Aβ plaques and NFTs, still recognized as the most representative AD brain hallmarks, adipose inclusions were originally observed by Alois Alzheimer in glial cells and described in 1907, suggesting a malfunctioning of lipid metabolism in the AD brain [99]. Alterations in the lipid composition have also been observed in post mortem AD brains, but a clear link between AD and lipid metabolism has been established with the identification of ApoE *ε*4 allele as a strong genetic risk factor [6]. In the last few decades, much evidence has supported the role of lipids, in particular cholesterol, in AD pathogenesis, and it is now clear that cholesterol homeostasis failure in the brain causes synaptic dysfunction and cognitive decline affecting neuronal functioning [5,100,101].

The brain is the most lipid-rich organ since it contains a quarter of the whole body’s non-esterified cholesterol pool [102,103]. Brain cholesterol metabolism is largely independent from that of peripheral tissues since plasma and brain cholesterol pools are separated by two barriers, the BBB and the blood-cerebrospinal fluid (CSF) barrier [104]. Maintaining proper cholesterol homeostasis in the brain is essential for neuronal functioning and brain development. In the adult brain most cholesterol is produced in situ: its synthesis occurs mainly in astrocytes and to a lesser extent in neurons. During embryogenesis, both neurons and glial cells synthesize cholesterol for myelinogenesis, but adult neurons gradually lose their synthetic ability and rely on astrocytes for cholesterol supply [105].

**Figure 2:**
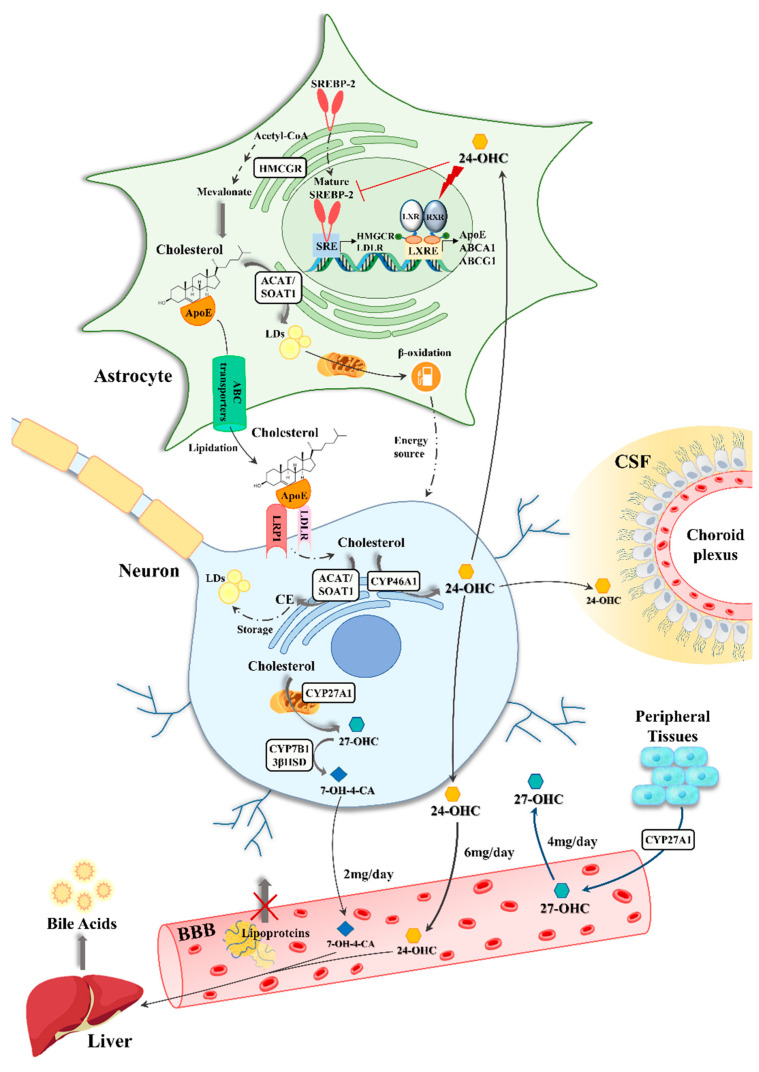
Brain cholesterol homeostasis. Abbreviations: 24-OHC, 24-hydroxycholesterol; 27-OHC, 27-hydroxycholesterol; 3βHSD, 3β-hydroxysteroid dehydrogenase; 7-OH-4-CA, 7α-hydroxy-3-oxo-4-cholestenoic acid; ABC, ATP-binding cassette; ACAT1/SOAT1, acyl-coenzyme A: cholesterol acyltransferase 1; ApoE, apolipoprotein E; BBB, blood-brain barrier; CE, cholesterol esters; CSF, cerebrospinal fluid; CYP27A1, cholesterol 27-hydroxylase; CYP46A1, cholesterol 24-hydroxylase; CYP7B1, oxysterol 7α-hydroxylase; HMGCR, 3-hydroxy-3-methyl-glutaryl-coenzyme A reductase; LDLR, low-density lipoproteins receptor; LDs, lipid droplets; LRP1, LDLR-related protein 1; LXR, liver X receptor; LXRE, LXR-responsive elements; RXR, retinoid X receptor; SREBPs, sterol regulatory element-binding proteins.

Cholesterol biosynthesis is a multistep process, expensive for cells in terms of energy. A well-depicted scheme that displays the most important steps of this pathway in the brain is shown in Figure 2. The first step occurs in the endoplasmic reticulum (ER), where acetyl-CoA is converted into 3-hydroxy-3-methylglutaryl CoA (HMG-CoA). Subsequently, HMG-CoA reductase catalyzes HMG-CoA conversion into mevalonate, which, after several reactions, is converted to lanosterol. After these common steps, depending on the cells in which it occurs, brain cholesterologenesis can take place by distinct pathways that produce cholesterol through different sterol intermediates: i) the Kandutsch–Russell pathway in neurons, in which cholesterol derives from 7-dehydrocholesterol (7D); and ii) the Bloch pathway in astrocytes, in which cholesterol derives from desmosterol (DE) [106]. Synthesized cholesterol is then rapidly transferred from the ER to the plasma membrane. Cholesterol synthesis is regulated by sterol regulatory element-binding protein 2 (SREBP-2), a transcription factor immobilized in the ER membrane when inactive. When cholesterol levels in the ER are low, the cholesterol detector SREBP cleavage-activating protein (Scap) binds to SREBP-2 and guides it into the Golgi compartment [107]; here, Scap releases the N-terminal domain of SREBP-2 which translocates into the nucleus and binds to sterol regulatory elements in the promoter regions of genes essential for cholesterol biosynthesis [108,109].

Cholesterol produced in the brain combines with ApoE, synthesized by astrocytes, to form lipoproteins secreted in the extracellular fluid through ABC transporters present in astrocyte membranes, and then transported to neurons [110,111,112]. In the CNS there are three isoforms of ABC transporters: ABCA1, ABCG1, and ABCG4. Among them, ABCA1 plays a major role in brain cholesterol homeostasis since it is involved in the lipidation and steady-state concentration of ApoE [113]. Its expression in astrocytes is lower than that in neurons; however, it represents the main type of ABCA transporters detected in astrocytes [114]. ABCA1 expression is mainly regulated by the transcription factors, liver X receptors (LXRs) and retinoid X receptors (RXRs), which bind to specific promoter regions of target genes when activated by specific ligands (i.e., oxysterols and DE) [115,116]. ABCA1 initiates the reverse cholesterol transfer in astrocytes by transferring cholesterol to unlipidated-ApoE. A second step of cholesterol transfer occurs via ABCG1 and leads to the formation of the so-called high-density lipoprotein (HDL)-like particles, spheroid lipidated-ApoE with density similar to that of circulating HDL. ApoE-containing lipoproteins are then taken up by two classes of LDL receptors, LDLRs and LRPs [117]. Although they are expressed in both astrocytes and neurons, LDLR is highly expressed in astrocytes, whereas LRP1 is mainly expressed in neurons [118]. Following receptor-mediated endocytosis, ApoE is recycled and cholesterol is used for cell membrane turnover and repair, myelin formation, synaptogenesis, and neurotransmitter release [102,103,119].

To maintain the steady-state levels, excess cholesterol is esterified by the enzyme acyl-coenzyme A: cholesterol acyltransferase 1 (ACAT1/SOAT1) in the ER and stored in LDs in the form of cholesterol esters [120]. ACAT/SOAT1 is more active in neurons than in glial cells; however, it becomes active in astrocytes under conditions such as the lack of ApoE or exogenous cholesterol overload [121]. Toxic fatty acids (FAs) produced in neurons are transferred to astrocytic LDs by ApoE-positive particles in order to protect neurons from FA toxicity during periods of hyperactivity, in which high levels of ROS induce FA peroxidation. Astrocytes consume the FAs stored in LDs via mitochondrial β-oxidation as an alternative energy source [122]. In addition, cholesterol can be complexed with ApoA1-containing lipoproteins and released directly into the CSF via ABC transporters, especially ABCA1 [123]. Last, but not least, excess cholesterol is mainly converted into the more hydrophilic metabolite 24-OHC by the neuron-specific enzyme 24-hydroxylase (CYP46A1) [124]. The levels of 24-OHC are directly correlated to cholesterol levels in the brain. Unlike cholesterol, this oxysterol diffuses across the BBB into the systemic circulation driven by the concentration gradient and then it is delivered to the liver for further degradation to bile acids [125,126]. A relevant percentage of 24-OHC in the body (80%) is produced and found in the brain [127], for this reason it is commonly called “cerebrosterol”.

In the brain, 24-OHC is involved in a regulatory loop between astrocytes and neurons that controls cholesterol homeostasis [117]. It regulates the cholesterol production by astrocytes by inhibiting the enzyme HMG-CoA reductase. Moreover, being a natural endogenous agonist of LXRs, 24-OHC up-regulates the expression of the LXR target genes coding for ABCA1, ABCG1 and ApoE, all key regulators of cellular cholesterol homeostasis, as demonstrated in primary astrocytes and in astrocytoma cells [128,129]. To a lesser extent, brain cholesterol is also oxidized to 27-OHC by sterol 27-hydroxylase (CYP27A1), that is expressed in neurons, astrocytes and oligodendrocytes at very low concentrations, and then into 7α-hydroxy-3-oxo-4-cholestenoic acid (7-OH-4-C) by the enzyme oxysterol 7-alpha-hydroxylase (CYP7B1); finally, 7-OH-4-C crosses the BBB and reaches the liver where it is eliminated [130,131,132]. However, most 27-OHC travels in the opposite direction by diffusion from the circulation into the brain through the BBB, since it is the major cholesterol metabolite in the circulation and the 27-hydroxylase is expressed in most of the organs and tissues [10]. Thus, along with the efflux of 24-OHC from the brain, the inflow of 27-OHC also occurs in the brain. The ratio 27-OHC:24-OHC is tightly regulated and it should remain constant in the different brain areas: for example, 1:8 in the frontal cortex, 1:5 in the occipital cortex and 1:10 in the basal ganglia [133]. Another oxysterol, present in the brain, of both enzymatic and non-enzymatic origin, is 25-hydroxycholesterol (25-OHC). It is produced enzymatically from cholesterol oxidation on its lateral chain by CYP27A1 and by the cholesterol 25-hydroxylase (CH25H) [134].

Certain oxysterols of enzymatic origin have been shown to be key regulators of cholesterol homeostasis in astrocytes since they suppress cholesterol biosynthesis in response to high cholesterol levels, as a sort of negative feedback. In particular, in a study performed in C6 glioma cells, 24-OHC and 27-OHC have been demonstrated to reduce free cholesterol and cholesterol ester content by inhibiting HMG-CoA reductase, SREBP1-a and LDLR expression in a dose-dependent manner [135]. Most likely, oxysterols block cholesterol synthesis by inhibiting the transport of SREBPs from the ER to the Golgi apparatus; in particular, they act by binding to insulin-induced gene proteins (Insig), favoring their binding to the SREBP-escort protein Scap [136]. Moreover, the oxysterols 24-OHC, 27-OHC and 25-OHC are able to induce the expression of ABCA1 and ABCG1 transporters in U-87 MG astrocytoma cells; among the three oxysterols, 24-OHC was found to be the most potent [137].

### 3.2. The Impact of Oxysterols on Astrocytes in Alzheimer’s Disease

Several oxysterols have been shown to accumulate in the brain in a toxic amount with AD development, in particular due to increased BBB permeability [138]. Besides the oxysterols of enzymatic origin (24-OHC, 25-OHC and 27-OHC), others deriving from cholesterol autoxidation are generated in the brain due to the high cerebral oxidative stress [139]. Indeed, the considerable brain oxygen consumption, the high neuronal metabolic rate and the low antioxidant defences make the brain particularly susceptible to oxidative stress. In support of this, accumulating evidence indicates that oxidative stress is an early event in AD pathogenesis [140]; a prominent increase of ROS, in fact, has been observed prior to the formation of amyloid plaques or NFTs [141,142,143]. As an early event in AD, oxidative stress has been shown to contribute to tau hyperphosphorylation and to Aβ oxidation in neuroblastoma cells [144,145], as well to affect glucose [146] and cholesterol metabolism [147]. In AD, oxidative stress and inflammation contribute to neurodegeneration in a sort of vicious circle: oxidative stress induces activation of astrocytes and microglia with the consequent release of pro-inflammatory molecules, and, in turn, glial activation leads to toxic radical release, exacerbating neuronal damage [2].

The concentrations of various oxysterols of enzymatic and non-enzymatic origin was examined post mortem in human AD brains at different stages of the disease. The systematic analysis was carried out on frontal and occipital cortices of AD subjects, classified by the Braak staging system of neurofibrillary pathology, and clarified the association between oxysterol levels in the brain and disease progression. In particular, the amounts of 27-OHC and of some oxysterols deriving from cholesterol autooxidation, including 25-OHC, 7-ketocholesterol (7-KC), 7α- and 7β-hydroxycholesterol (7α-, 7β-OHC), α- and β-epoxycholesterol (α-, β-epoxy), and 4α- and 4β-hydroxycholesterol (4α-, 4β-OHC), were found to be significantly increased in the brains. Conversely, 24-OHC content was found to be markedly decreased in the later stages, and its loss is likely a factor in accelerating AD development. Of note is that expression levels of the CYP46A1 gene decreased in parallel with 24-OHC, and the levels of the CYP27A1 gene increased with AD progression, reflecting the increasing trend of 27-OHC levels [139]. The decrement of both CYP46A1 and 24-OHC in the late stages is probably due to the selective loss of neurons expressing CYP46A1. In the normal brain, this enzyme is expressed in both neurons and astrocytes, but in AD the levels of 24-OHC decrease mainly because of the neuronal damage; however, in AD there is an ectopic induction of CYP46A1 in astrocytes, especially around senile plaques, that leads to some 24-OHC production but without compensating for its decrease [148]. As regards the marked increment of CYP27A1 and 27-OHC in the end-phases of AD, there are different explanations. Acknowledging the fact that CYP27A1 expression is considerable in neurons, its expression, and consequently 27-OHC levels, might decrease due to neuronal death; however, 27-OHC levels rise markedly over the course of AD because CYP27A1 is also expressed in astrocytes and microglia, potentially leading to in situ generation of the oxysterol [131]. In addition, in cases of hypercholesterolemia, the excessive production of 27-OHC in the periphery leads to increased flow of 27-OHC to the brain across the damaged and impaired BBB [125]; a further cause may be the reduced expression of CYP7B, the neuronal enzyme responsible for 27-OHC metabolism, because of neuron loss [149].

The association between altered cholesterol metabolism and AD progression has been evident for many years [2,132,150]. Indeed, oxysterols present in the brain cause neuron dysfunction and degeneration by enhancing oxidative stress and inflammation [2]; however, some of them, including 24-OHC, have recently been shown to exert neuroprotective effects [151,152].

**Figure 3:**
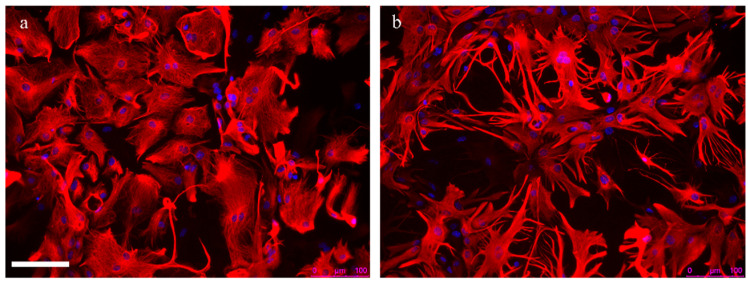
The morphological change induced by oxysterols in mouse primary astrocytes. Control astrocytes (**a**) and astrocytes treated with an oxysterol mixture representative of oxysterols present in late AD brains (**b**) ([67], unpublished images). Scale bar: 100 µm.

The effects of oxysterols on neurons have been widely demonstrated, though there are still few data regarding their role in astrocytes during AD. Given the presence of numerous reactive astrocytes in AD, a recent study deepened understanding of the role of oxysterols in modulating astrocyte reactivity. Oxysterol mixtures (10 µM), representative of oxysterols present in early or late stages of AD brains, were shown to induce a similar and clear morphological change in mouse primary astrocytes. The marked change in astrocyte morphology induced by oxysterols, characterized by the appearance of long and branched processes, is shown in Figure 3 [78] (unpublished images). Moreover, the two oxysterol mixtures, and particularly that characteristic of the late AD stages, were able to up-regulate some reactive astrocyte mediators, including Lcn2, cytokines and chemokines, that affect neuronal health and synapses. It has also been demonstrated that oxysterol-activated astrocytes induce synaptotoxicity mediated by Lcn2, as shown by the marked decrease of the number of dendritic spines and by the reduced complexity of neurites in primary neurons co-cultured with astrocytes [78].

It has been demonstrated that 27-OHC (5–20 µM) is deleterious to C6 glioma cells since it induces oxidative stress and up-regulates the nuclear factor erythroid 2-related factor 2 (Nrf2) antioxidant response leading to cell death [153]. In addition, 24-OHC affects redox homeostasis in human U-87 MG astrocytoma cells depending on its concentration: at low concentrations (1 or 5 µM) it contributes to maintaining redox homeostasis by reducing ROS production, while, at high concentrations (10 or 20 µM) it leads to an increase of ROS release, likely dependent on fall in the antioxidant defense [154]. Moreover, high concentrations of 7β-OHC (50 µM) and, to a lower extent, of 7-KC (50 µM) have been shown to exert cytotoxic effects, both on cell viability and cell growth, in C6 glioma cells and in mixed glial murine primary cultures (astrocytes and oligodendrocytes), as well as in 158N murine oligodendrocytes and in SK-N-BE neuroblastoma cells [155]. High concentrations of 7β-OHC (30 µM) have been shown to induce a morphological change characterized by processes elongation and to induce cytotoxicity in rat primary astrocytes with increased cAMP levels, which are considered a valid in vitro model of reactive astrocytes [156]. Contrasting results have indicated that the injection of liposomes containing 7β-OHC in the hippocampus of rats previously injected with iron reduces the extent of reactive astrogliosis by lowering GFAP expression [157].

Another very recent study highlighted the detrimental effects of excess 27-OHC in the brain due to plasma hypercholesterolemia. Loera-Valencia and colleagues demonstrated that 27-OHC enhances, in primary rat neurons and astrocytes, an inflammatory signaling cascade by triggering a response mediated by alarmin involving the receptor for advanced glycation end products (RAGE) and its ligand S100A8 [158].

Epstein–Barr virus-induced G protein-coupled receptor 2 (EBI2) is a G-protein-coupled receptor required for humoral immune responses, and one of the membrane receptors that bind oxysterols. In particular, 7α,25-dihydroxycholesterol (7α,25-OHC) was identified as a potent and selective agonist of EBI2. Activation of EBI2 by 7α,25-OHC, and closely related oxysterols, has been shown to induce B cell migration and regulate T-cell-dependent antibody response, thus linking oxysterols to the adaptive immune response [159]. The EBI2 receptor is also expressed by human and mouse astrocytes, where it has recently been demonstrated that the 7α,25-OHC/EBI2 signaling mitigates the response to pro-inflammatory signals in vitro and limits the levels of brain pro-inflammatory cytokines in vivo [160].

In the last few years, the close relationship between cholesterol metabolism, glucose uptake, and the renin-angiotensin system (RAS), all of which are affected in neurodegenerative diseases, has been highlighted in the brain [119]. In this connection, excess 27-OHC in the brain is considered a biomarker for reduced brain glucose metabolism in AD since it increases brain RAS activity, thus impairing glucose uptake by neurons. Indeed, CYP27A1 over-expressing mice showed decreased glucose metabolism and memory deficits [161]. The treatment of primary astrocytes and neurons with both 27-OHC or 24-OHC (1 µM) regulated the brain RAS through an LXR-dependent mechanism by stimulating the production of angiotensinogen, angiotensin I-converting enzyme (ACE) and angiotensin II type 1 receptors, all involved in neuronal plasticity, learning and memory [162,163].

Finally, it has been demonstrated that the brain lipid profile may be modified by the long-term exposure to a high-fat diet (HFD) [164]. In particular, the levels of 27-OHC, transported from the peripheral circulation, increased in high-cholesterol fed rabbits causing degeneration in the hippocampus [165]. Importantly, to support the impact of cholesterol dysmetabolism in astrocyte reactivity, the HFD triggers astrocytic activation associated with increased expression of functional markers including ApoE and aquaporin 4, and with the release of IL-1β in the murine hippocampus [166].

### 3.3. The Role of ApoE4 Astrocytes in Alzheimer’s Disease

The *ε4* allele of ApoE has been identified as a major risk factor for late-onset AD [6]. In addition to its role in the periphery, ApoE is the main apolipoprotein responsible for lipid/cholesterol transport in the CNS, where it is mainly produced by astrocytes [167]. Three ApoE isoforms are present in humans (ApoE2, ApoE3, and ApoE4) and AD frequency in *ε4* homozygotes is much higher (91%) compared to *ε3* homozygotes (20%) [6]. A single amino acid change characterizes ApoE2 (Cys112, Cys158) and ApoE4 (Arg112, Arg158) compared to the most prevalent isoform ApoE3 (Cys112, Arg158); the presence of Arg112 in ApoE4 leads to the change of the ApoE structure, that affects its ability to bind lipids, receptors, and Aβ [168]. Concerning brain implications, several features have been observed in ApoE4 carriers compared to non-carriers: increased Aβ deposition [169,170], reduced Aβ clearance in the CSF [170,171], grater brain atrophy in subjects affected by AD [172], reduced dendritic spine density in the hippocampus [173], as well as a higher risk of developing cerebral amyloid angiopathy [174], and of progression from mild cognitive impairment to AD [175]. In order to study the impact of human ApoE isoforms in vivo, ApoE-targeted-replacement mice (ApoE-TR mice), in which the mouse ApoE gene has been replaced with the human ApoE gene, have been created. ApoE4-TR mice are characterized by Aβ and hyperphosphorylated tau accumulation [176], reduced dendritic spine density in the entorhinal cortex, deficits in synaptic transmission [177], and cognitive impairment [176,178]. An increase in Aβ plaques and a reduction of its clearance in the CSF have been observed also in other mouse models expressing ApoE4 [171,179,180,181]. Animal models, human-induced pluripotent stem cells (hiPSCs), and CRISPR-Cas9 genome editing, have enabled deeper understanding of the impact of ApoE4 in different CNS cell types, including astrocytes [182]. Several mechanisms through which ApoE4 may increase the risk of developing AD have been suggested, including both Aβ-dependent and -independent effects [183].

As previously described, ApoE [67,184] and its receptors LDLR and LRP1 [66,68] have been shown to play a key role in astrocyte-mediated Aβ clearance, though the mechanisms underlying the impact of the ApoE genotype on Aβ levels remains to be elucidated. Inducible and astrocyte-specific expression of human ApoE4 in APP/PS1 mice led to increased amyloid deposition and associated dystrophic neurites when ApoE4 expression was induced in the early seeding stage of amyloid pathology (0–6 months) [185]. Moreover, hiPSC-derived ApoE4 astrocytes, compared to ApoE3 astrocytes, have been shown to produce and secrete lower levels of ApoE, as well as to have a reduced ability to clear Aβ, probably due to a deficit in lysosome-mediated Aβ degradation [186]. Moreover, Prasad and colleagues showed that the endosomal Na^+^/H^+^ exchanger 6 is down-regulated in mouse astrocytes expressing human ApoE4, leading to an excessive endosomal acidification that traps LRP1 in the endocytic compartment and causes a subsequent reduction of Aβ clearance [187]. Though studies have shown ApoE ability to bind Aβ [188] and reduced Aβ clearance in ApoE KO astrocytes [67], it has been observed that ApoE affects Aβ uptake because of its competitive binding to LRP1 and LDLR, rather than directly binding Aβ [184]. Furthermore, lipoprotein particles released by ApoE4 astrocytes have been found to be hypolipidated [189,190], a feature that has been shown to affect ApoE affinity for Aβ [189,191]. It has been suggested that poor ApoE4 lipidation could be due to its ability to affect ABCA1 membrane trafficking. Indeed, astrocytes from ApoE4-TR mice showed lower levels of membrane ABCA1 because of increased levels of ADP-ribosylation factor 6 that traps ABCA1 in the late-endosomes, leading to lipid-poor ApoE4 particles and to a deficit in Aβ clearance; this was retrieved by treatment with the ABCA1 agonist CS-6253 [192]. As discussed by Tai and colleagues, contrasting results about the formation of ApoE/Aβ complexes could be due to different methods of detection, the lipidation status of ApoE, and the ApoE:Aβ ratio used to perform experiments; moreover, the study of ApoE4 lipidation status is limited by the difficulties in isolating and analyzing intact CNS lipoproteins [193]. However, treatment of APP/PS1 mice with bexarotene, a RXR agonist, showed a significant reduction of Aβ plaques in the cortex and hippocampus, increased expression of ApoE and cholesterol transporters ABCA1 and ABCG1, increase in HDL release, and the restoration of cognitive and memory functions [69]. The reduction of Aβ levels in the brain interstitial fluid was not observed in ApoE KO mice, and in vitro experiments on primary astrocytes and microglia confirmed that the bexarotene effect on Aβ degradation depends on pathways regulating ApoE levels and on ApoE itself [69].

Among Aβ-independent effects, the impact of ApoE genotype on brain lipid homeostasis is an increasingly studied topic. The transcriptomic analysis of hiPSC-derived astrocytes has revealed that genes involved in lipid metabolism are differentially expressed in ApoE3 and ApoE4 astrocytes, with the latter also showing higher amounts of intracellular cholesterol [186]. Indeed, lower levels of ABCA1 in the membrane of ApoE4 astrocytes derived from ApoE-TR mice were also associated with a reduced cholesterol efflux, which was restored by treating astrocytes with the ABCA1 agonist CS-6253 [192]. ApoE4 astrocytes have also been shown to accumulate more and smaller LDs, not due to increased FA uptake but probably due to reduced FA degradation [194,195]. In addition, these cells showed reduced efficiency in mobilizing neuronal LDs and in taking up LDLs, as well as reduced energetic and synaptic support to neurons; importantly, these deficits in neuron-astrocyte coupling of FA metabolism have been shown to be the cause of the compromised metabolic support [195]. Furthermore, ex vivo analysis of hippocampal brain slices from ApoE4-TR mice further validated the results obtained in astrocytes, showing a shift towards glucose metabolism in ApoE4 brains likely due to defective FA oxidation [195]. In addition, hiPSC-derived ApoE4 astrocytes have been shown to secrete hypolipidated lipoproteins and to be less effective in promoting neuronal survival and synaptogenesis when co-cultured with neurons, further confirming the substantial impact of compromised astrocytic metabolic support on neuronal health [190]. Moreover, it has recently been observed that astrocytic ApoE is able to regulate cholesterol synthesis and histone acetylation in neurons by delivering microRNA (miRNAs). ApoE4-containing lipoproteins have been shown to contain lower amounts of specific miRNAs, thus leading to a reduced ability to regulate neuronal cholesterol synthesis and the transcription of genes involved in memory consolidation [196].

Another interesting question, that is still debated, is the influence of ApoE genotype on astrocyte reactivity. Despite human AD brain analysis finding no significant differences between ApoE4 carriers and non-carriers concerning the number of reactive astrocytes [51], studies on ApoE4-TR mice and AD mouse models carrying the human ApoE4 isoform showed increase in reactive astrocytes and microglia [181,185,197], as well as increased levels of pro-inflammatory cytokines and greater synaptic protein loss, after LPS injection [181,197]. Interestingly, reactive astrocytes secrete higher levels of ApoE-containing lipoproteins, and long-chain saturated free FA have been shown to play a role in mediating the neurotoxic effects of reactive astrocytes [41].

Several ApoE-based therapeutic approaches (e.g., regulation of ApoE levels or lipidation, blocking ApoE interaction with Aβ, and ApoE mimetics) have been demonstrated to be effective in AD mouse models. However, only a few human clinical trials have been completed or are in the process, and they have shown very limited effects [198]; therefore, further studies are needed to deepen understanding of the role of ApoE4 in AD, in order to identify the best therapeutic approach for targeting ApoE.

## 4. Conclusions

Impaired cholesterol metabolism and astrocyte reactivity in the brain are key factors in AD onset and progression. Indeed, the idea that targeting astrocytes [199,200,201] or maintaining cholesterol homeostasis [152,198] could represent new potential therapeutic strategies to inhibit or delay AD progression is gaining ground. However, understanding of their mutual influence needs to be further developed, in particular whether and how brain cholesterol dysmetabolism affects astrocyte reactivity and vice versa.
